# The Role of CCL24 in Systemic Sclerosis

**DOI:** 10.5041/RMMJ.10504

**Published:** 2023-07-31

**Authors:** Hilit Levy, Udi Gluschnaider, Alexandra Balbir-Gurman

**Affiliations:** 1 R&D, Chemomab Ltd, Tel Aviv, Israel; 2 Rheumatology Institute, Rambam Health Care Campus, Haifa, Israel; 3 Ruth and Bruce Rappaport Faculty of Medicine, Technion–Israel Institute of Technology, Haifa, Israel

**Keywords:** CCL24, CCR3, chemokine, eotaxin-2, systemic sclerosis

## Abstract

Systemic sclerosis (SSc) is a chronic immune-mediated disease characterized by microangiopathy, immune dysregulation, and progressive fibrosis of the skin and internal organs. Though not fully understood, the pathogenesis of SSc is dominated by microvascular injury, endothelial dysregulation, and immune response that are thought to be associated with fibroblast activation and related fibrogenesis. Among the main clinical subsets, diffuse SSc (dSSc) is a progressive form with rapid and disseminated skin thickening accompanied by internal organ fibrosis and dysfunction. Despite recent advances and multiple randomized clinical trials in early dSSc patients, an effective disease-modifying treatment for progressive skin fibrosis is still missing, and there is a crucial need to identify new targets for therapeutic intervention. Eotaxin-2 (CCL24) is a chemokine secreted by immune cells and epithelial cells, which promotes trafficking of immune cells and activation of pro-fibrotic cells through CCR3 receptor binding. Higher levels of CCL24 and CCR3 were found in the skin and sera of patients with SSc compared with healthy controls; elevated levels of CCL24 and CCR3 were associated with fibrosis and predictive of greater lung function deterioration. Growing evidence supports the potency of a CCL24-blocking antibody as an anti-inflammatory and anti-fibrotic modulating agent in multiple preclinical models that involve liver, skin, and lung inflammation and fibrosis. This review highlights the role of CCL24 in orchestrating immune, vascular, and fibrotic pathways, and the potential of CCL24 inhibition as a novel treatment for SSc.

## INTRODUCTION

### Disease Manifestations and Clinical Impact

Systemic sclerosis (SSc) is a complex autoimmune rheumatic disease, characterized by immune system dysregulation, vascular alterations, and widespread fibrosis, resulting in skin thickening and organ damage including lungs, peripheral blood vessels, kidneys, heart, and digestive system.^1^,^2^ Dissemination of skin fibrosis in the periphery is defined as limited SSc. Approximately 70% of individuals diagnosed with SSc will exhibit this specific disease subset. The presence of skin thickening in both peripheral and central areas is indicative of diffuse skin involvement (dSSc). The etiology of SSc is unknown; the pathogenesis of the disease is complex and depends on its specific subset and stage (early or late). It was suggested that the fibrotic process is a result of initial vascular endothelial insult, immune system activation followed by autoantibody formation, and excessive collagen and extracellular matrix production by fibroblasts and their activated form, myofibroblasts.^3^ Morbidity and mortality rates are higher in SSc compared to other connective tissue diseases, with a 3-year survival rate of 47%–56%, particularly in patients with target organ complications such as interstitial lung disease (ILD), cardiac involvement, pulmonary arterial hypertension, and scleroderma renal crisis.^4^–^6^ Severity and distribution of skin fibrosis correlate with vital organ involvement, digital ulcers, quality of life, and SSc-related mortality. In patients with dSSc, internal organ damage occurs very early and progresses rapidly. Therefore, initiation of treatment early in the disease course is essential to slow disease progression and attenuate long-term complications. There is significant clinical heterogeneity between SSc patients, reflected by diverse patterns of skin and organ involvement evident in different disease stages, different sexes and ethnic groups, as well as divergences between auto-antibodies profile, such as antinuclear antibodies, antibodies to topoisomerase, to centromere, to RNA polymerase III, and others.^1^

### Current Treatment Challenges

The regulation of fibrosis in SSc is a highly intricate process involving both innate and adaptive immune processes. This includes the activation of various cell types such as macrophages, plasmacytoid dendritic cells, mast cells, monocytes, as well as B and T lymphocytes. Fibroblasts and myofibroblasts are also significant players in the fibrotic milieu characteristic of scleroderma.^7^ The complexity of SSc was recently demonstrated by Gur et al. in a comprehensive single-cell RNA sequencing study, evaluating the blood and skin samples from SSc patients. The study showed that changes in the immune cell compartment were primarily observed in subsets of dSSc patients, corresponding to different SSc skin pathology phases (early inflammatory phase with immune activation versus fibrotic phase). For the first time obvious differences between blood and skin components were demonstrated. Patients with severe dSSc exhibited a significant reduction in the number of scleroderma-specific fibroblasts, which correlated with SSc severity.^8^ Cellular activation in SSc is associated with excessive production of pro-inflammatory and pro-fibrotic cytokines, chemokines, and growth factors, including interleukin (IL)-1β, IL-6, IL-4, IL-10, tumor necrosis factor-α, tissue growth factor-β, CCL18, CXCL4, toll like receptor-4, interferons type 1 and 2, and others.^9^

The complex course of SSc, along with the various phenotypes and patterns of skin and organ involvement, demands a comprehensive approach. With growing knowledge on SSc pathogenetic pathways, the use of biological drugs targeting anti-inflammatory and anti-fibrotic mechanisms has been proposed. Unfortunately, most randomized controlled trials (RCTs) that have utilized biologicals to target skin fibrosis in dSSc have failed to achieve their primary endpoint. Several open and randomized open studies have reported on the positive effect of cyclophosphamide (CYC) on scleroderma skin, and two small RCTs have shown a modest effect of methotrexate on skin fibrosis in early dSSc.^9^ A phase II RCT investigating the effectiveness of abatacept, a CD80/CD86 blocker that inhibits the interaction with CD28 T cells in early dSSc, did not reach its primary endpoint of reducing modified Rodnan skin score; nevertheless, secondary outcome measures, including gene expression subsets, showed evidence in support of abatacept in skin biopsies from patients with inflammatory or normal-like skin.^9^,^10^ In a phase II RCT, romilkimab, a humanized IgG4-neutralizing antibody that blocks IL-4/IL-13, demonstrated efficacy in early dSSc. However, while these findings are promising, a phase III trial is necessary as previous candidate treatments for dSSc, which showed promising results in phase II trials, ultimately failed to demonstrate treatment benefits in phase III trials.^9^,^11^,^12^

Notwithstanding the above, to date there is still no United States Food and Drug Administration (FDA)-approved treatment for skin involvement in dSSc. In contrast, the organ-based approach, focusing on treatment directed to stabilize lung function in SSc-ILD, has yielded more success, as evidenced by the positive results of the scleroderma lung study-I, scleroderma lung study-II, and SENSCIS RCTs.^13^–^15^ Altogether, the wide spectrum of disease manifestations and multi-organ involvement in SSc highlights the necessity for disease-modifying therapies that align with the evolving understanding of the pathophysiology of this condition.

### Emerging Therapeutic Approaches

The concept of using immunosuppressants to treat SSc-ILD and expecting improvement in additional clinical domains in SSc is not new. A comparison of safety and efficacy between CYC and mycophenolate mofetil treatment in scleroderma lung studies revealed improvement in skin condition measured by modified Rodnan skin score.^16^ In recent years, two drugs with different mechanism of action, nintedanib (tyrosine kinase inhibitor) and tocilizumab (anti IL-6 receptor blocker), have been approved by the FDA for the treatment of SSc-ILD.^7^,^14^ The efficacy of nintedanib is based on well-established data on the key pathogenetic pathways of pulmonary fibrosis in general and in SSc in particular. Nintedanib inhibits the main pro-fibrotic targets in pulmonary fibrosis: vascular endothelial growth factor receptor, platelet-derived growth factor receptor, and fibroblast growth factor receptor.^7^,^14^ However, it should be noted that nintedanib did not show any effect on skin thickening.

It is widely recognized that IL-6 plays a significant role in triggering inflammatory conditions. Blockade of IL-6 receptor by tocilizumab was effective in several rheumatic diseases and was approved by the FDA for the treatment of rheumatoid arthritis (RA), giant cell arteritis, and systemic juvenile idiopathic arthritis. Studies have also shown the involvement of IL-6 in fibrosis. Patients with SSc have shown high levels of IL-6 in their serum, and these levels correlated with more prominent skin involvement. In addition, increased secretion of IL-6 from skin and lung biopsy samples of SSc patients has been shown to be associated with the proliferation and accumulation of B lymphocytes, particularly during the early stages of the disease (within 6 years of diagnosis).^17^–^20^ Based on these findings, a phase III RCT with tocilizumab was conducted in patients with early dSSc and increased levels of inflammatory markers.^21^,^22^ Although the primary endpoint regarding skin involvement was not achieved in this study, treatment with tocilizumab in patients with early dSSc did stabilize the pulmonary condition as measured by forced vital capacity.^22^

Involvement of B lymphocytes in SSc pathogenesis advocated the use of targeting CD20 in SSc, particularly given its effectiveness in other rheumatic diseases such as RA, vasculitis, and IgG4 disease. Recently, results were reported of the RECITAL RCT comparing rituximab (an anti-CD20 mAb) with CYC in patients with ILD related to connective tissue diseases, including SSc. Both drugs were found to be similarly effective in terms of ILD improvement. Rituximab had preferable safety profile compared to CYC. However, data on any potential skin changes observed during this study have not been published yet.^23^ In an open randomized trial (DESIRES), rituximab was effective in treating patients with early dSSc and ILD in both domains: lungs and skin.^24^ Radical treatment including CYC and rescue bone marrow transplantation showed better results in skin and lung function improvement and event-free survival than did standard treatment with CYC; the treatment was associated with relatively high procedure-related morbidity and mortality and today is used in selected patients with rapidly progressive skin involvement in the absence of heart and kidney involvement.^25^–^27^

Indeed, the available treatment solutions for skin and lung fibrosis in SSc are not yet optimal. In this regard, different molecular pathways are currently under clinical evaluation as possible therapeutic targets in SSc. These include drugs targeting fibrotic pathways as well as drugs targeting the persistent activation of the immune system.^12^,^28^ Given the heterogeneity of the pathological pathways implicated in SSc onset and progression, new treatment strategies for SSc should ideally target multiple pathways, including immune-mediated inflammation, vasculopathy, and fibrosis.^12^,^28^ This review aims to provide an overview of the existing treatment modalities targeting different pathogenetic pathways in SSc with particular emphasis on the significance of CCL24 in SSc pathogenesis, focusing on its impact on both the immune and stromal components. Inhibition of CCL24 may offer a multi-faceted approach for SSc treatment, by targeting the main key SSc processes, the vasculopathy, inflammatory, and fibrotic triad.

## CHEMOKINES INVOLVED IN THE PATHOLOGY OF SSC

Chemokines are a group of secreted proteins within the cytokine family, that have small molecular weight (8–13 kilodaltons) and are categorized into four different families (CC, CXC, CX3C, C) based on the presence of NH_2_ terminal cysteine motifs. These small signaling proteins that induce migration and activation of cells are key players in orchestrating the influx of immune cells into disease-target organs and driving inflammatory responses following specific triggers.^29^,^30^

The chemokine-receptor network is complex; altogether, 50 chemokines and 20 receptors have been identified in humans.^31^ Chemokines and their receptors have been implicated in multiple inflammatory diseases, such as atherosclerosis, multiple sclerosis, psoriasis, insulin resistance, and more.^32^–^36^ The initiation and progression of SSc involves multiple chemokines and inflammatory cells such as T cells, macrophages, dendritic cells, eosinophils, B lymphocytes, fibroblasts, and mast cells.^37^ They have also been shown to play a significant role in the pathogenesis of SSc, fostering migration and activation of inflammatory cells, promoting the transformation of fibroblasts into myofibroblasts, and impairing angiogenesis and vasculogenesis in SSc patients.^37^–^40^

## CCL24—A MULTIFACTORIAL CHEMOKINE

Eotaxins belong to a CC chemokine subfamily that plays a role in chemotaxis. There are three members in this family: eotaxin-1 (CCL11), eotaxin-2 (CCL24), and eotaxin-3 (CCL26). Although all three eotaxins engage to the same cognate receptor, CCR3, they were shown to induce recruitment and/or activation of different cells depending on specific pathological conditions.^41^–^43^ The chemokine CCL24 promotes cell trafficking exclusively via the CCR3 receptor.^44^ It induces chemotaxis and activation of CCR3-expressing cells, including eosinophils,^45^–^47^ basophils,^48^ T cells,^49^ fibroblasts,^50^ and monocytes.^51^ Besides, CCL24 is produced by activated immune cells, predominantly M2 macrophages, as well as by activated epithelial cells, and it is known to play a role in various inflammatory, fibrotic, and vascular processes.^52^–^55^

### CCL24 as a Pro-inflammatory Chemokine

The pro-inflammatory function of CCL24 was demonstrated in several preclinical models. In a murine model of ovalbumin-induced pulmonary inflammation, CCL24 knockout mice exhibited reduced cell infiltration into the bronchoalveolar lavage fluid, indicating a significant decrease in inflammation.^56^ Similar results were also evident in a bleomycin-induced skin-and-lung inflammation and fibrosis mouse model, using CCL24 knockout mice.^57^ This suggests that CCL24 contributes to the inflammatory response seen in these models, highlighting its pro-inflammatory function in the context of pulmonary inflammation.

Clinical observations have revealed that CCL24 levels are associated with disease progression in various inflammatory conditions. In patients with asthma, increased expression of CCL24 in the bronchial tissue has been correlated with eosinophilic inflammation following allergen challenge.^58^ In patients with RA, elevated levels of CCL24 were detected in synovial fluids.^59^ Moreover, an exaggerated inflammatory response involving CCL24 in the airways has been identified as a predictor of disease deterioration in patients following SARS-CoV-2 infection.^60^

### CCL24 as a Pro-fibrotic Chemokine

Beyond its proinflammatory activity, CCL24 was also found to play a role in the induction of profibrotic effects. Kohan et al. previously showed that CCL24 induced proliferation of human lung fibroblasts and collagen synthesis *in vitro*, and to be constitutively expressed by dermal fibroblasts.^52^,^61^ Its cognate receptor, CCR3, was demonstrated to be expressed on dermal fibroblasts where it modulated wound healing and tissue remodeling.^62^,^63^

In patients, CCL24 demonstrated prominent expression and activity in various inflammatory-fibrotic diseases. In liver diseases that involve fibrosis, significant overexpression of CCL24 and CCR3 was found in liver biopsies and blood samples from patients with fibrotic non-viral chronic liver diseases such as non-alcoholic fatty liver disease (NAFLD) and non-alcoholic steatohepatitis (NASH) compared to healthy individuals.^64^ Moreover, Lin et al. showed that CCL24 levels are significantly increased in the sera of patients with primary biliary cholangitis.^65^ Interestingly, CCL24 levels in the lung were found to be associated with severity and progression of idiopathic pulmonary fibrosis (IPF), a disease sharing many similar clinical features with SSc-associated ILD.^66^,^67^

### CCL24 Involvement in Angiogenesis

Further to the inflammatory and profibrotic activity promoted by CCL24, it was also shown to be involved in angiogenesis-related pathways.^68^ Several studies have demonstrated that CCL24 may affect endothelial cell tube formation, migration, and invasion features.^54^ These effects are driven by specific mediators including toll-like receptor 4 and VEGF-A.^54^,^69^ Also, CCL24 signaling was shown to play a role in the tumor microenvironment in various types of cancer including colon cancer, hepatocellular carcinoma, and cutaneous T cell lymphoma.^70^

### CCL24 Involvement in SSc

The accumulating data supporting the CCL24 role in inflammation and fibrosis paved the way to evaluate its involvement in SSc. The expression pattern of CCL24 in SSc was evaluated in patients’ skin and serum samples. Mor et al. demonstrated that CCL24 levels were elevated in the sera of SSc patients compared to healthy controls, and it was overexpressed in the skin of SSc patients along with its receptor.^57^ Additionally, in SSc patients with dSSc, serum levels of CCL24 positively correlated with fibrosis biomarkers and deterioration of lung function over time.^71^ A study by Lee et al. also revealed that monocyte populations isolated from SSc patients showed overexpression of CCR3, the cognate receptor for CCL24.^72^ Furthermore, CCL24 was shown to promote pathways associated with type 2 immune response, involving Th2 lymphocytes and M2 macrophage transformation, both of which have been implicated in fibrosis, including in SSc.^73^,^74^ These findings suggest that CCL24 may play a role in the pathogenesis of SSc, particularly in relation to fibrosis and immune dysregulation, and could serve as a potential target for therapeutic intervention.

## CCL24 AS A THERAPEUTIC TARGET

The novel monoclonal antibody CM-101 is designed to bind and neutralize CCL24 activity. It was evaluated in various *in vitro*, *in vivo*, and *ex vivo* preclinical models and was shown to significantly interfere with fibrotic and inflammatory key pathways.

The anti-inflammatory effect of CCL24 inhibition was shown across multiple inflammatory animal models. In an experimental model for atherosclerosis using ApoE knockout mice, blocking antibodies to CCL24 resulted in a significant reduction of early atherosclerotic plaques, and prolonged treatment of mice with advanced plaques led to atheroma stabilization.^75^ In an animal model for RA, namely adjuvant-induced arthritis (AIA), significant inhibition of arthritis was observed in rats treated with anti-CCL24 antibodies compared to control.^76^ Additionally, in a model for multiple sclerosis, treatment of experimental autoimmune encephalomyelitis-induced mice with anti-CCL24 antibody significantly decreased symptom severity.^77^

The activity of CM-101 was then evaluated in diseases that involve both inflammation and fibrosis in the liver, skin, and/or lung. Treatment with CM-101 in experimental liver fibrosis-related animal models resulted in a significant reduction of liver collagen deposition and inflammatory mediated factors. This reduced liver damage was also seen in CCL24 knockout mice, which further validates the relevance of CCL24 as a primary target in inflammation and fibrosis.^64^ Moreover, *in vitro* studies further supported the *in vivo* findings. Data showed that inhibiting CCL24 resulted in the attenuation of liver fibroblast migration and activation, as demonstrated by reduced alpha-smooth muscle actin (α-SMA) expression and secretion of pro-collagen 1.^64^

**Figure 1:**
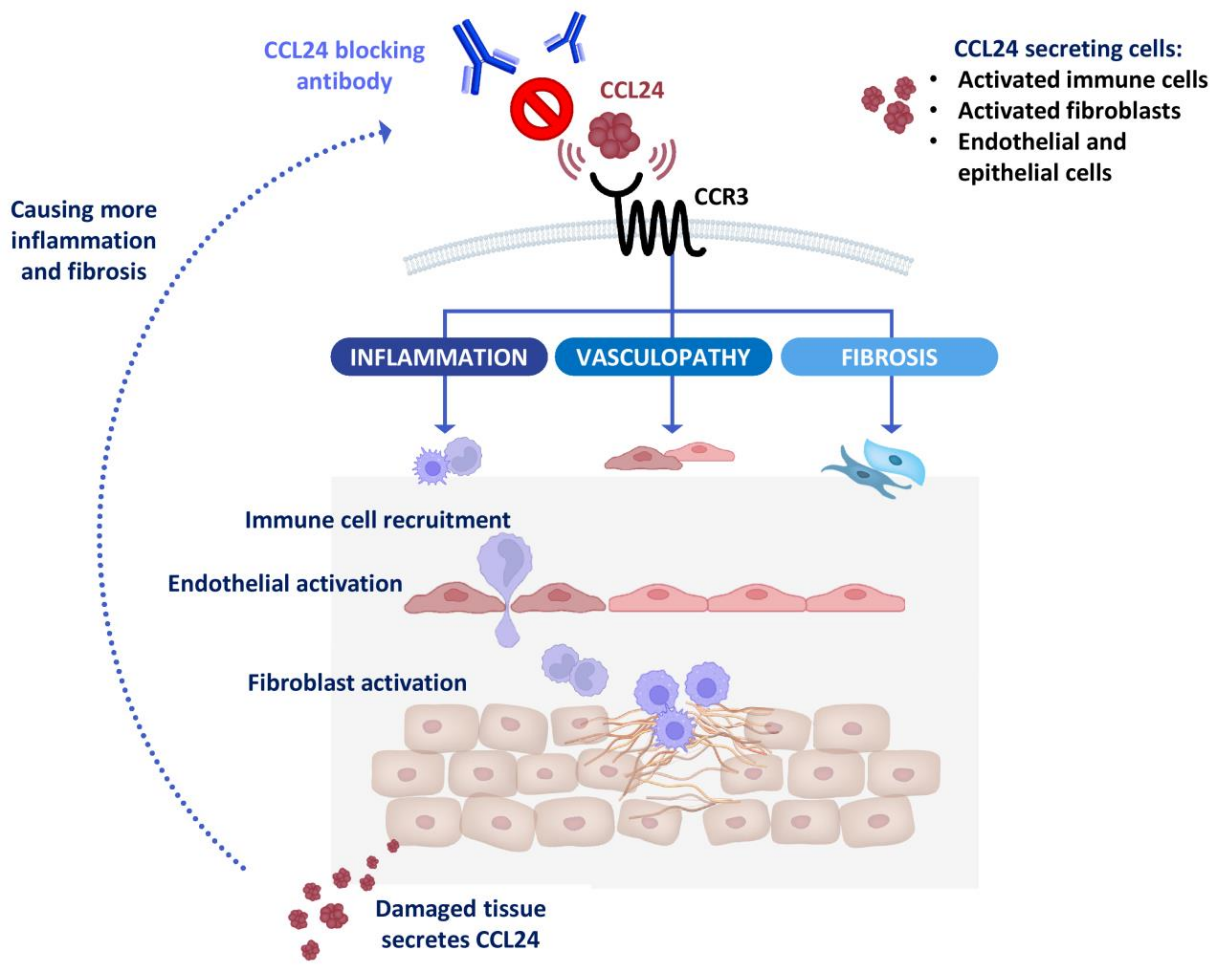
Mechanism of CCL24 Involvement in SSc Pathology. Following CCL24 binding to CCR3, immune cells recruitment, dermal fibroblast activation, and vascular impairment are seen. Secretion of CCL24 is elevated in the fibrotic tissue resulting in further recruitment of immune cells, increased vascular damage, and additional fibroblasts activation, which, eventually, results in additional secretion of CCL24. Neutralization of CCL24 with CM-101 abrogates its binding to CCR3 and breaks the “vicious cycle” by attenuating inflammation and fibrosis.

Multiple preclinical models highlighted the effect of CM-101 on skin and lung inflammation and fibrosis. It was shown, using both preventive and therapeutic approach in a bleomycin-induced skin and lung fibrosis model in mice, that CCL24 blockade resulted in significant inhibition of collagen deposition, reduction in dermal thickness, and decreased infiltration of immune cells to the lung. *In vitro*, CM-101 was shown to attenuate migration and activation of dermal fibroblasts and to inhibit the activation of endothelial cells.^57^ Additional studies also showed that silencing CCL24 using shRNA affected tube formation, which further supports CCL24-related effects on vasculopathy.^54^,^57^ Taken together, the preclinical data highlight the important role of CCL24 in inflammation, fibrosis, and vasculopathy, suggesting that CCL24 could be a potential therapeutic target in SSc as well as in other fibrotic inflammatory diseases (Figure 1).

## CONCLUSIONS

Systemic sclerosis is a complex, immune-mediated disease with prominent microangiopathy and fibrosis, associated with substantial morbidity and mortality. Despite the advances in drug development in many aspects of rheumatic diseases over the past years, disease-modifying drugs are still needed in SSc. The chemokine CCL24 is implicated in the pathogenesis of SSc via pro-inflammatory, vascular, and pro-fibrotic pathways. Treatment with CCL24-neutralizing antibody (CM-101) in preclinical models of SSc and other inflammation and fibrosis-related models has shown robust efficacy in attenuating inflammation and fibrosis. The data underscore the importance to study CM-101 in patients with SSc.

## Abbreviations

CCL11: eotaxin-1
CCL24: eotaxin-2
CCL26: eotaxin-3
CYC: cyclophosphamide
dSSc: diffuse systemic sclerosis
FDA: United States Food and Drug Administration
IL: interleukin
ILD: interstitial lung disease
RA: rheumatoid arthritis
RCT(s): randomized controlled trial(s)
SSc: systemic sclerosis
